# A complete mitochondrial DNA genome of whitefly species (Hemiptera: Aleyrodidae) from *Litchi chinensis*

**DOI:** 10.1080/23802359.2018.1551076

**Published:** 2019-07-26

**Authors:** Hua-Ling Wang, Teng Lei, Yin-Quan Liu

**Affiliations:** a Ministry of Agriculture Key Laboratory of Molecular Biology of Crop Pathogens and Insects, Institute of Insect Sciences,Zhejiang University, Hangzhou, China;; b Natural Resources Institute, University of Greenwich, Kent, United Kingdom

**Keywords:** Mitochondrial genome, phylogeny, whitefly, *Litchi chinensis*

## Abstract

A novel complete mitochondrial genome (mitogenome) of whitefly species, collected from *Litchi chinensis* at Fujian province of China (hereafter whitefly_*Litchi chinensis* _China) (GenBank accession number: MH999477), was described in this study. The mitogenome of whitefly_*Litchi chinensis* _China is 15,360 bp in length and contains 13 protein-coding genes, 21 transfer RNAs, 2 ribosomal RNAs and a non-coding AT-rich region (D-loop). The arrangement of mitochondrial genes of whitefly_*Litchi chinensis*_China are identical with *Aleurochiton aceris*, but remarkably different from the mitogenomes of the other whitefly genus. Most protein-coding genes (PCGs) start with ATN, except for *nad2*, *cox2* and *atp6* genes starting with TTG, GTG, and TTG, respectively; 10 of the 13 PCGs use the typical stop codon TAN, whereas *cox1*, and *cox2* stop with a single T. Phylogenetic analyses based on 13 PCGs support the close relationship of the sample with *Aleurochiton aceris*, which would provide us further insights on the taxonomy and phylogeny of Aleyrodidae.

Whiteflies (Order Hemiptera, Family Aleyrodidae) can be considered as the second most important vectors due to their capacity to transmit many plant viruses (España and López-Moya 2014) and comprise more than 1,156 species in 126 genera (Mound and Halsey 1978). However, the whitefly species of many countries of the world are poorly known. For the whitefly systematics, it is in veritable disarray because variable pupal morphology can be altered by environmental factors which have been found to be common in many genera, especially in the subfamily Aleyrodinae (Neal and Bentz 1999). Consequently, most whitefly species in the Aleyrodinae have been arbitrarily placed in various genera over the years with no clear idea of their phylogenetic relationships (Russell 1957; Mound 1963; Mound and Halsey 1978; Gill 2012). Such instance drive taxonomists to seek the proper molecular markers for aiding inferring the systematics and evolutionary history of whitefly.

To enrich the whitefly molecular markers, in this study, a newly complete mitogenome of whitefly belonging to the subfamily Aleyrodinae collected from Lichi at Fujian province, China (hereafter Whitefly_*Litchi chinensis* _China) was determined. The sample was deposited at the Key Laboratory of Agricultural Entomology, Institute of Insect Science, Zhejiang University, Hangzhou, China. The total genomic DNA was extracted from a single individual using the Qiagen DNeasy Blood and Tissue Kit Extraction Kit (Germany) (Wang et al. 2013). The DNA was then subjected to conduct next-generation sequencing which generated 20GB raw pair-end reads (Illumina HisSeq 2500; 2*150bp, Shanghai, China). The clean reads were assembled by NOVOPlasty software (Dierckxsens et al. 2017) with setting up available whitefly mitogenomes as references. The resultant contigs were annotated using softwares of Geneious (Drummond et al. 2011), tRNAscan-SE (Lowe and Eddy 1997) and website of MITOS (Bernt et al. 2013).

The whole mitochondrial genome is a circular molecule of 15,360 bp (30.98% A, 43.61% T, 14.84% G, and 11.02% C) in size, and contains 13 PCGs, 21 tRNA genes (t-RNA-Glu is absent), and two rRNA genes and a non-coding AT-rich region (D-loop) (GenBank accession number: MH999477). A 1,536 bp of the *cox1* gene shows 77% sequence similarity to a *cox1* sequence of *Aleurochiton aceris* in GenBank (accession number AY572538.1). Remarkedly, the gene arrangement and orientation are identical with the *Aleurochiton aceris* but differ from the other whitefly species. Most of PCGs start with ATN, except for *nad2*, *cox2* and *atp6* genes starting with TTG, GTG, and TTG, respectively; 10 of the 13 PCGs use the typical stop codon TAN, whereas two PCGs (*cox1* and *cox2*) stop with the incomplete codon T.

In addition, with the maximum-likelihood (ML) and Bayesian inference (BI) methods through RAxML (version 8.2.4) (Stamatakis 2006) and MrBayes (version 3.2.6) (Ronquist and Huelsenbeck 2003), phylogenetic trees were constructed using 13 PCGs of mitogenomes from the closely related whitefly species. In the commands of MrBayes and RAxML, the data were partitioned by codons based on the partition schemes derived from PartitionFinder 2 (Lanfear et al. 2016). The resultant ML and BI trees share the same topologies and show that our specimen (whitefly_ *Litchi chinensis* _China _MH999477) cluster together with *Aleurochiton aceris* (Figure 1).

**Figure 1.**
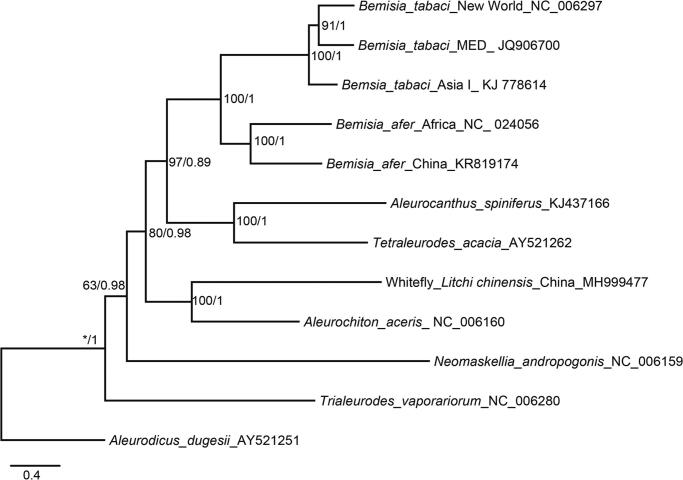
Maximum likelihood (ML) and Bayesian inference (BI) phylogenetic trees inferred from the nucleotide sequence data of mitogenomic 13 PCGs. *100/1.00(BP/BPP)
